# A Rare Case of a Gigantic Retroperitoneal Schwannoma

**DOI:** 10.3390/medicina60081203

**Published:** 2024-07-25

**Authors:** Nenad Koruga, Borna Kovačić, Alen Rončević, Branko Dmitrović, Zrinka Požgain, Anamarija Soldo Koruga, Tatjana Rotim, Sonja Škiljić, Hrvoje Vinković, Tajana Turk

**Affiliations:** 1 Department of Neurosurgery, University Hospital Center Osijek, 31000 Osijek, Croatia; 2 Faculty of Medicine, Josip Juraj Strossmayer University of Osijek, 31000 Osijek, Croatia; 3 Department of Abdominal Surgery, University Hospital Centre Osijek, J. Huttlera 4, 31000 Osijek, Croatia; 4 Department of Pathology and Forensic Medicine, University Hospital Center Osijek, 31000 Osijek, Croatia; 5 Faculty of Dental Medicine and Health, Josip Juraj Strossmayer University of Osijek, 31000 Osijek, Croatia; 6 Department of Neurology, University Hospital Center Osijek, 31000 Osijek, Croatia; 7 Department of Diagnostic and Interventional Radiology, University Hospital Center Osijek, 31000 Osijek, Croatia; 8 Department of Anesthesiology and Critical Care, University Hospital Center Osijek, 31000 Osijek, Croatia

**Keywords:** schwannoma, abdominal neoplasms, retroperitoneal space, somatosensory evoked potentials

## Abstract

*Introduction*: Schwannomas (Schs) are benign tumor masses that rarely occur intra-abdominally and rarely reach larger diameters. When present, they occur as rare solitary nerve sheath tumors of peri-neural Schwann cells. Schwannoma mostly affects the nerves of the extremities, trunk, or the head and neck region. They are more common in female patients, mostly among patients between the third and fifth decade. They occur spontaneously but could also be found in association with a group of genetic autosomal dominant disorders called type 2. When present intra-abdominally, schwannomas grow slowly without significant clinical signs and symptoms. Clinical importance is presented in cases of occupying intra-abdominal space and impingement of surrounding structures, which causes intermittent pain. Only 0.5–5% of all retroperitoneal tumors are schwannomas and their malignant transformation is very rare. *Case report*: The authors present a case of a large intra-abdominal schwannoma in a 70-year-old female patient. She underwent CT scanning due to refractory left-sided subcostal pain, which revealed a large tumor mass in the left-sided hemiabdomen. Preoperative cytologic biopsy confirmed Sch. The patient underwent an MRI scan upon admission to our department, which revealed the origin of the tumor at the left-sided L3 level and intra-abdominal tumor spreading with the largest diameter of 25 cm. The patient underwent multidisciplinary surgical excision, confirmed by MRI scan in a period of five months postoperatively. *Conclusions*: Its rare presentation leads to the necessity to adequately evaluate such patients, especially to avoid any hidden diagnosis which might lead to further complications. The goal of a multidisciplinary approach should be emphasized as maintaining a good postsurgical condition without neurological deficits.

## 1. Introduction

Schwannomas (Schs) or neurilemmomas are generally benign tumors which affect patients between the second and the fifth decade twice as frequently in female patients compared to male patients [1]. The primary origin of Schs is Schwann cells, which are found in all cranial and peripheral nerves. Only three percent arise in the peritoneum, where they grow enormously prior to its diagnosis and subsequently cause clinical difficulties as a consequence of their size; also, Schs represent only up to 5% of all retroperitoneal tumors [2,3]. Therefore, compared to other anatomical sites, retroperitoneal schwannomas (RS) are more likely to experience spontaneous bleeding and degeneration [3]. The World Health Organization excluded the term “malignant schwannoma”; nevertheless, its malignant form might develop as malignant peripheral nerve sheath tumors (MPNST) [4]. An early diagnosis is especially important for the malignant form of Schs since they are high-grade sarcomas that are extremely malignant, intensely invasive, prone to recurrence and metastasis, and have a low long-term survival rate [5]. Schwannomas are found in the genetic autosomal dominant disorder called schwannomatosis [6].

The typically, pathological appearance consists of spindle cells with elongated nuclei and an alternation of Antoni A (hypercellular) and Antoni B (hypocellular) patterns; some cases may also exhibit verocay bodies as an alternation of nuclear palisades with fibrillar regions. Malignant neoplasms such as synovial sarcoma or leiomyosarcoma are represented by Antoni A areas that do not exhibit nuclear palisading. The S-100 protein presents robust and diffuse positivity in the IHC analysis of these malignancies [7]. Schs might be found in association with genetic autosomal dominant disorders such as neurofibromatosis type 2 [8]. 

The authors present a rare case of surgically treated gigantic retroperitoneal Schwannoma causing symptoms consistent with its gigantic presentation.

## 2. Case Report

**Figure 1:**
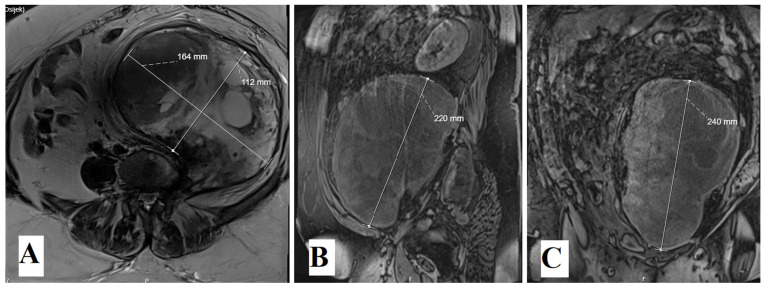
Preoperative enhanced T1-weighted MRI scans revealed large intraabdominal tumor in all three projections: axial, sagittal and coronal (**A**–**C**).

The patient was admitted to our department due to the obscure left-sided costal arch pain which lasted for three months prior to admission. Due to refractory pain, the patient underwent a computed tomography (CT) scanning of the abdomen, which revealed a large tumor mass which occupies the left hemiabdomen, compresses the diaphragm and visceral intrabdominal organs to the contralateral side. The smallest portion of the tumor is located at the level L3 and compresses the nerve root and neural foramen. Magnetic resonance (MR) imaging confirmed a large solid mass with the largest diameter of 25 cm and its origin at the left-sided L3 root. MRI was performed upon admission to our department (Figure 1).

According to her medical history, the patient underwent cytologic tissue sampling, which confirmed Sch. The patient is a regular smoker with no significant comorbidities except arterial hypertension and obesity. 

During the preoperative evaluation of the patient, no significant deviations were found, including laboratory findings. The preoperative physical examination of the patient included a muscle strength test of the left lower limb, which suggested a full range of antigravity movements with some resistance (grade 3+). Limited flexion and rotation in the lumbosacral region were noted, the left lower limb straight leg raising test (Lasegue) revealed motion of more than 35 degrees. The patient reported no significant pain and the test was mostly limited by obesity. Sphincter control was fully preserved.

**Figure 2:**
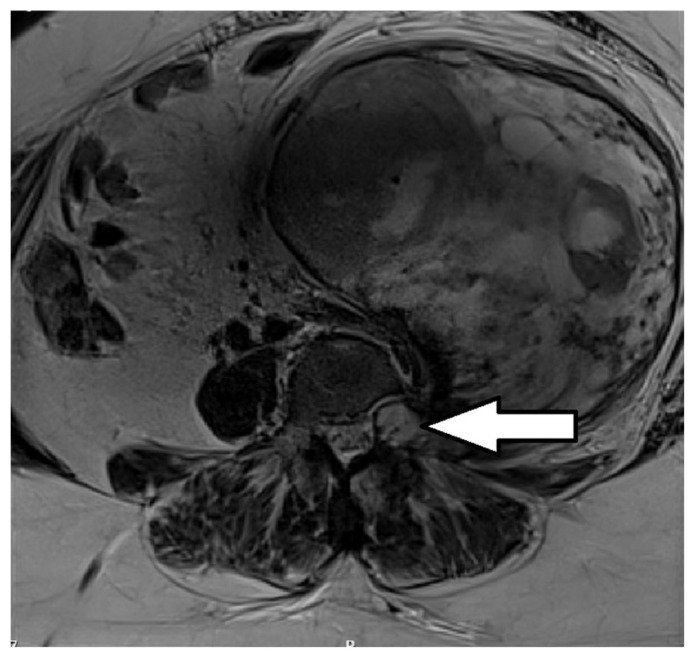
Preoperative axial T1-weighted enhanced MRI scan revealed tumor origin at the level of the right-sided L3 foramen (arrow).

Surgical technique: Surgery was performed multidisciplinary, in two stages. The original idea was to de-attach the Sch from its root at the level of L3 (Figure 2). According to MRI, the authors believed that such a procedure could have preserved the L3 root and facilitate further transabdominal resection of the tumor with decreased risk of the nerve injury. 

Therefore, the first stage in general anesthesia included neurosurgical approach to the lumbar spine. We performed the left-sided interlaminectomy at the level of L3 with a far-lateral approach. The tumor appeared as a whitish, solid mass. The nerve root was completely encased and there were no visible limits which would indicate tissue boundaries. Deattachment of the tumor from the osseal neural foramen was carried out carefully with respect to healthy anatomical boundaries using a high-speed drill, excochleation surgical instrument and rarely bipolar cauterization. After hemostasis, wound closure went uneventfully in a regular fashion. 

**Figure 3:**
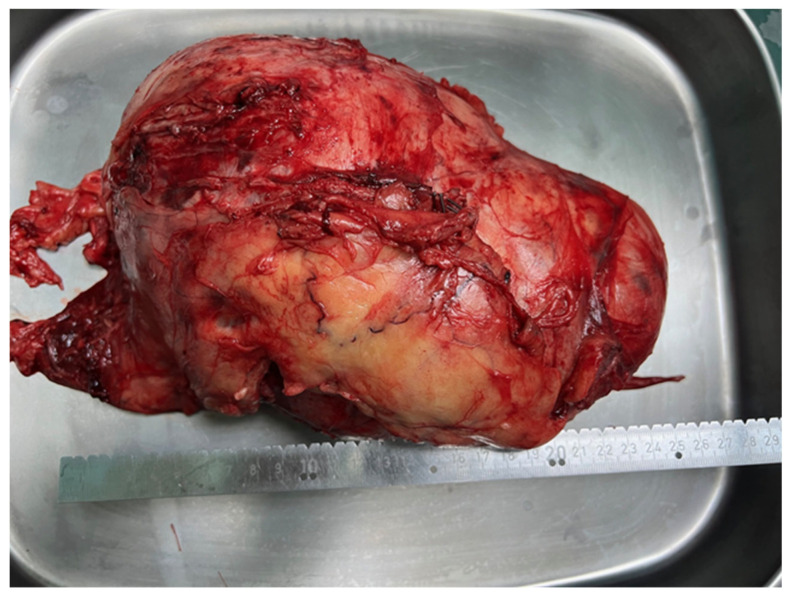
Postoperatively extracted tumor, diameter measurement of 25 cm measured by scale.

Abdominal surgeons performed a median laparotomy as a second, consequent stage of surgery. After a meticulous approach, a large retroperitoneal tumor was presented in the entire left hemiabdomen. It had pushed the descending colon, left ureter and vascular structures towards the medial and right hemiabdomen. The tumor was completely removed and separated from the spinal column to which it was attached with solid adhesions (Figure 3). Verriset hemostatic patch (Medtronic) was applied at the tumor origin site after its resection. The drain tube was also placed at the retroperitoneal space with no consequent bleeding during the postoperative recovery of the patient. 

**Figure 4:**
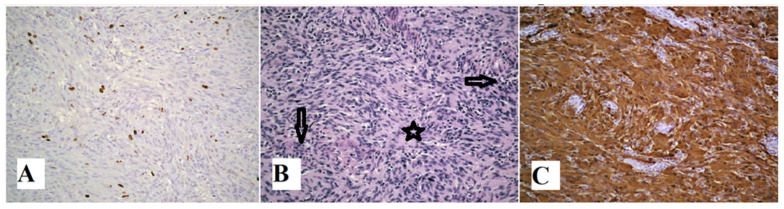
(**A**) Low nuclear Ki-67 immunoreactivity (×200) indicating low tumor cell proliferation rate. (**B**) Neoplasm composed of cytologically bland spindle cells arranged in intersecting fascicles with nuclear palisading. No histologic features of malignancy are identified. (H&E, ×200). Arrows indicate Antoni A, densely packed cells, and star presents Antoni B area with loosely organized cells. (**C**) S100 immunohistochemical stain (×200) of surgically resected tumor showed strongly positive diffuse nuclear and cytoplasmic staining for S-100 protein.

An early postoperative period was complicated by the partial lumbar wound dehiscence; the patient was obese and was lying on her back due to a large laparatomy wound. Ten days after surgery, we revised and re-sutured the wound, and antibiotic treatment was introduced. Meanwhile, pathohistological analysis revealed the diagnosis of schwannoma, low nuclear Ki-67 immunoreactivity, which delineated low tumor cell proliferation rate, cytologically bland spindle cells arranged in intersecting fascicles with nuclear palisading, and S100 immunohistochemical stain, which revealed strongly positive diffuse nuclear and cytoplasmic staining for S-100 protein (Figure 4). 

**Figure 5:**
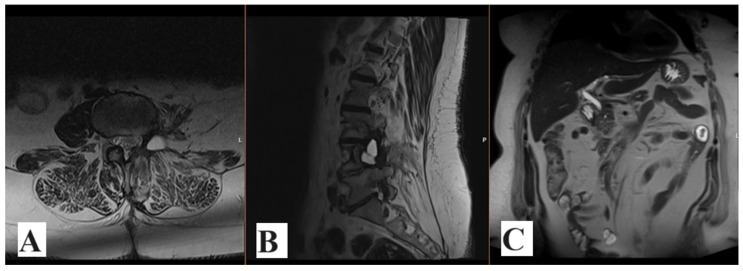
Axial (**A**), sagittal (**B**) and coronal (**C**) postoperative T2-weighted MRI scan of the lumbar spine and abdomen revealed no tumor recurrence or remnants at the level of the tumor origin.

The follow-up MRI scan five months after surgery revealed an excellent postoperative outcome with no tumor relapse nor tumor remnants presented (Figure 5). The patient also underwent somatosensory evoked potentials (SEP) examination for the tibial nerve, which detected normal recordings for the stimulated nerve. Nevertheless, electromyography (EMG) revealed total denervation of the left-sided L3 root. The patient is self-ambulatory with mild paresthesias down the left leg; physical therapy was proposed after the hospital discharge. 

## 3. Discussion

The most common sites of Schwannomas (Schs) are extremities, head and neck, and their presentation in the abdomen is a rare finding, with only up to 3% occurring in the retroperitoneum [9]. 

Schwannomas are benign tumors arising from the peripheral nerve sheaths and their malignant forms are rare findings. When malignant forms are presented, their clinical presentation is manifested as infiltrating and intraneural spreading as well as proliferating and herniating forms, mostly into the vessels. Malignant forms of Schs are rare and their symptoms depend on the anatomical site of its origin; also, malignant forms usually lead to the more complicated symptoms such as intestinal, pelvic and perineal disorders. Extraintestinal forms of Schs are more often benign and commonly lead to non-specific symptoms related to their size and consequent compression of surrounding structures. In such cases of extraintestinal Sch, its diagnosis is usually delayed due to paucity of symptoms, as presented in our case. Also, these tumors are well encapsulated with cystic degenerations and calcifications [10]. 

Symptoms in our patient were obscure, as expected in such slowly proliferating tumors, and a preoperative biopsy confirmed a benign Sch, which was finally confirmed postoperatively by histopathological exam. Diagnostic tools are of great aid in planning the future steps of treating such tumors; ultrasonography is the first option, followed by CT scans, which were utilized in pre-planning in our case. MRI scan is a method of choice in distinguishing the soft tissues; nevertheless, despite its advances, one cannot determine the right nature of the neoplasm. What is more advanced in using an MRI scan is the possibility of preoperative planning in terms of other peritumoral surrounding soft tissues. 

The diagnosis of Sch depends on immunohistochemistry and histopathologic exam with the recognition of the Antoni A and Antoni B areas which represent the histologic hallmark of Sch. In comparison, Antoni B regions are dominantly hypocellular and weakly organized, while Antoni A areas appear more organized. The majority of retroperitoneal lesions are encapsulated and S-100 protein positive [11]. Schs are resistant to radiation and chemotherapy; therefore, surgical resection presents the first-line treatment. Due to their benign nature, there are very small chances of tumor recurrence following total surgical removal and the prognosis is excellent [12]. 

The most appropriate approach in our case was lumbar detachment and consequent median laparatomy in order to achieve the best possible surgical result. Besides the removal of the tumor itself, it is necessary to achieve negative margins due to the possibility of tumor attachment to the surrounding anatomical structures and to avoid a possible tumor recurrence; therefore, further follow-up is necessary in order to recognize and avoid a possible onset of tumor recurrence or remnants.

Also, the quality of life in such patients depends on aforementioned criteria of how wide and safe surgical resection is. It is necessary to avoid intra-surgical damage of neural structures, such as the nerve roots, and to keep the patient neurologically intact. Nevertheless, sometimes the size of the tumor itself and neural encasement dictates the surgical approach, the limits of surgical resection and consequent postoperative deficits.

## Data Availability

Data are contained within the article.
